# Versatile Functionalization of Carbon Nanomaterials by Ferrate(VI)

**DOI:** 10.1007/s40820-019-0353-2

**Published:** 2020-01-21

**Authors:** Ying Zhou, Zhao-Yang Zhang, Xianhui Huang, Jiantong Li, Tao Li

**Affiliations:** 1 School of Chemistry and Chemical Engineering, Key Laboratory of Thin Film and Microfabrication (Ministry of Education), Shanghai Jiao Tong University, Shanghai, 200240 People’s Republic of China; 2 School of Electrical Engineering and Computer Science, KTH Royal Institute of Technology, Electrum 229, 16440 Kista, Sweden

**Keywords:** Ferrate(VI), Reactivity, Carbon nanomaterials, Oxidation

## Abstract

As a high-valent iron compound with Fe in the highest accessible oxidation state, ferrate(VI) brings unique opportunities for a number of areas where chemical oxidation is essential. Recently, it is emerging as a novel oxidizing agent for materials chemistry, especially for the oxidation of carbon materials. However, the reported reactivity in liquid phase (H_2_SO_4_ medium) is confusing, which ranges from aggressive to moderate, and even incompetent. Meanwhile, the solid-state reactivity underlying the “dry” chemistry of ferrate(VI) remains poorly understood. Herein, we scrutinize the reactivity of K_2_FeO_4_ using fullerene C_60_ and various nanocarbons as substrates. The results unravel a modest reactivity in liquid phase that only oxidizes the active defects on carbon surface and a powerful oxidizing ability in solid state that can open the inert C=C bonds in carbon lattice. We also discuss respective benefit and limitation of the wet and dry approaches. Our work provides a rational understanding on the oxidizing ability of ferrate(VI) and can guide its application in functionalization/transformation of carbons and also other kinds of materials.

**Electronic supplementary material:**

The online version of this article (10.1007/s40820-019-0353-2) contains supplementary material, which is available to authorized users.

## Highlights

- Various forms of carbon nanomaterials are selected as substrates to clear the mist in understanding the reactivity/utility of ferrate(VI) in oxidizing carbon nanomaterials.
- It unravels a modest reactivity of ferrate(VI) in liquid phase that only oxidizes the active defects on carbon surface and a powerful oxidizing ability in solid state that can open the inert C=C bonds in carbon lattice.
- Respective benefit and limitation of the wet and dry approaches using ferrate(VI) in functionalizing carbon nanomaterials are discussed.

**Electronic supplementary material:**

The online version of this article (10.1007/s40820-019-0353-2) contains supplementary material, which is available to authorized users.

## Graphical abstract

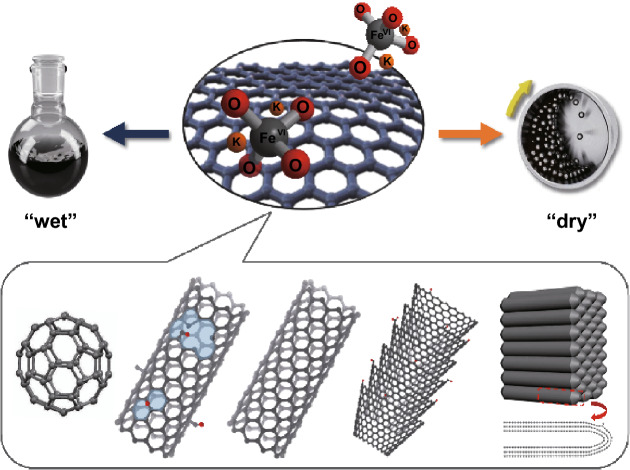

## Introduction

Nature utilizes Fe^IV ^=O and Fe^V^=O complexes as the active centers for a number of important enzymatic oxidations, which motivates many fundamental studies on the properties of high-valent iron compounds, especially their chemical and biological reactivities [1–8]. Fe^VI^, the highest accessible oxidation level of iron, generally exists in the form of ferrate(VI) ion (FeO_4_^2−^) with four Fe^VI^=O bonds. Ferrate(VI) possesses powerful oxidizing ability, as revealed by its higher redox potentials (up to + 2.2 V in acidic conditions) than those of most traditional oxidants [9–12]. Together with the environmentally benign nature, ferrate(VI) compounds (commonly K_2_FeO_4_) have been considered as promising oxidizing agents in several areas, including water remediation [13–17], organic synthesis [18–21], high-capacity battery [22–24] and O_2_ evolution [25–29].

Recent years have witnessed an emerging role of ferrate(VI) in materials science [30–34], where ferrate(VI)-enabled oxidative functionalization/transformation of a target material is a key step toward functional applications. Of particular interest is the oxidation treatment of carbon materials [35–41]. Peng et al. [35] reported the first example of applying K_2_FeO_4_ for the oxidation/exfoliation of graphite in concentrated H_2_SO_4_. Highly water-soluble single-layer graphene oxide was produced in 1 h at room temperature, which indicated the extraordinary oxidizing ability of K_2_FeO_4_ in H_2_SO_4_ medium. But soon after, Sofer and coworkers [36] provided completely opposite results, showing such liquid-phase oxidation was impossible for graphite, attributed to the extreme instability of K_2_FeO_4_ in acidic environments. Nevertheless, some other studies suggested the moderate oxidation effects of K_2_FeO_4_ by the production of graphite/graphene oxides with relatively low degrees of oxidation [37, 38] and by the result of nondestructive oxidation of carbon nanotubes (CNTs) [39]. Therefore, the reactivity of K_2_FeO_4_ in liquid phase (i.e., H_2_SO_4_ medium) remains confusing in oxidizing carbon materials, which ranges from aggressive to moderate, and even incompetent (see Table S1 for a comparison of the literature results).

Oxidations by ferrate(VI) under solvent-free condition provide an alternative and green way for harnessing its oxidizing power. Very recently, our group discovered that the intrinsic high reactivity of K_2_FeO_4_ was accessible in the “dry” solid state by oxidizing small-molecule substrates [42]. On this basis, K_2_FeO_4_ solids were applied for the oxidation of CNTs under mechanical milling, and effective surface functionalization was achieved. However, the dry chemistry of ferrate(VI) is still poorly understood and the mechanism of K_2_FeO_4_ oxidation on carbon materials remains unclear. A critical question is whether K_2_FeO_4_ is reactive enough to open the inert C=C bonds in carbon lattices.

**Fig. 1:**
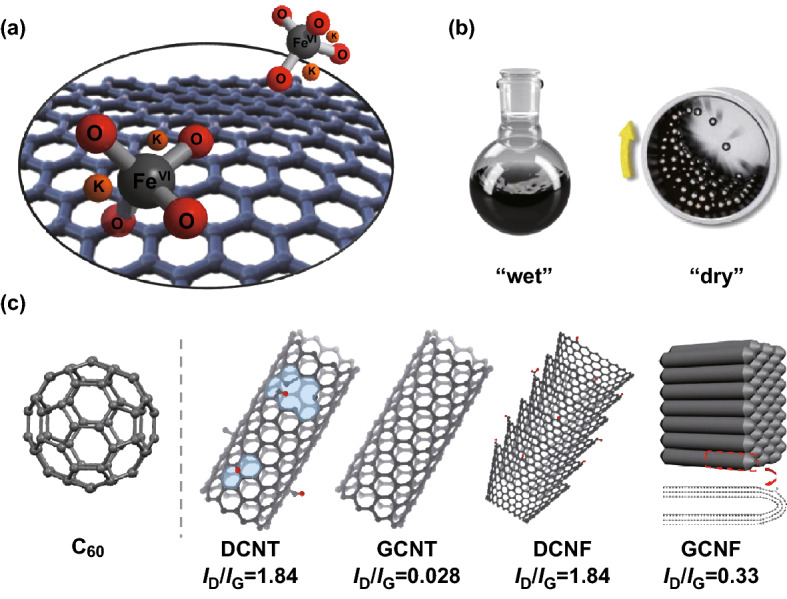
**a** A schematic representation of K_2_FeO_4_–carbon reaction system. **b** Depiction of two reaction conditions (in H_2_SO_4_ medium or by solid-state ball milling). **c** Structural models of the carbon nanomaterials used in this study: C_60_, defective CNT (DCNT), graphitized CNT (GCNT), defective and graphitized carbon nanofibers (DCNF and GCNF). *I*_D_/*I*_G_ refers to the relative intensity of D to G band in Raman spectrum, taken as a measure of defect degree

Herein, we scrutinize the reactivity of ferrate(VI) in liquid phase and solid state using various forms of carbon materials as substrates, as depicted in Fig. 1. Fullerene C_60_ is selected as a probe molecule, and four typical nanocarbons with diverse physical structures and different defect degrees are further used to test the reactivity. Our systematic results provide a rational understanding on the performance of this attractive oxidizer in materials chemistry.

## Experimental Section

### Preparation of K_2_FeO_4_

The purity of K_2_FeO_4_ should be examined before it is used for oxidizing carbon materials. The commercially supplied K_2_FeO_4_ products from several manufactures only have actual purities of 20% or below, although the declared purities are > 90%. Therefore, we synthesized K_2_FeO_4_ according to the literature [43] and purified them by recrystallization as described in our previous work [42]. Preparation details were provided in Supporting Information (SI).

### Oxidation of Carbon Materials

#### Liquid-Phase K_2_FeO_4_ Oxidation

Oxidation of C_60_. 30 mg of C_60_ was slowly added to 20.0 mL of sulfuric acid (95–98%) in a 50-mL two-necked flask under argon atmosphere, and the dispersion was sonicated for 30 min. Then 2.5 g of K_2_FeO_4_ was slowly added to the flask under argon flow at 0 °C, and the reaction mixture was stirred at 60 °C for 12 h. The resulting dispersion was diluted in 500 mL of cold water and stirred for 30 min. The solid was obtained by centrifugation (12,000 rpm), followed by washing in sequence with 2 M HCl (several times to remove Fe^3+^), ultrapure water (18.2 MΩ cm) and ethanol. The product was finally dried at 60 °C in a vacuum oven.

Liquid-phase oxidation treatments of other carbon materials were described in SI.

#### Solid-State K_2_FeO_4_ Oxidation

Oxidation of C_60_. 100 mg of C_60_ and 2.5 g of K_2_FeO_4_ were mixed together by brief grinding in an agate mortar. The mixture was then introduced into a 50-mL stainless milling jar together with 26 g of 5-mm-diameter stainless steel balls (ball-to-powder weight ratio 10:1). Ball milling was performed at a rotational speed of 250 rpm for 6 h or 180 rpm for 2 h in a horizontal planetary ball milling (WXQM-2L, Tecan Powder). The jar was opened every 30 min to break up the mixture materials if they were agglomerated or adhered to the sidewall during milling process. The obtained solid was washed by centrifugation (12,000 rpm) in sequence with 2 M HCl (several times to remove Fe^3+^), ultrapure water (18.2 MΩ cm) and ethanol. The product was finally dried at 60 °C in a vacuum oven.

Solid-state oxidation treatments of other carbon materials were described in SI.

### Characterization

The purity of K_2_FeO_4_ sample was tested by spectrophotometry, X-ray diffraction (XRD) and ^57^Fe Mössbauer spectroscopy (^57^Co(Pd) source). Chemical structure of C_60_ samples was determined using matrix-assisted laser desorption/ionization Fourier transform ion cyclotron resonance mass spectrum (MALDI-FTICR MS) and ^1^H NMR (400 MHz). Oxidation degrees of carbon materials were analyzed using X-ray photoelectron spectroscopy (XPS, binding energies were calibrated with respect to C 1s peak at 284.6 eV) and thermogravimetry (TG, 10 °C min^−1^, N_2_). Defect degree and morphology were characterized by Raman spectra, scanning electron microscopy and transmission electron microscopy (SEM and TEM). Details of instruments and test conditions were described in SI.

## Results and Discussion

### Purity Analysis of K_2_FeO_4_

K_2_FeO_4_ oxidizer used in our experiments has a purity of 95%, as determined by spectrophotometry. XRD pattern confirmed single-phase character of the solid (Fig. S1), and the ^57^Fe Mössbauer spectrum proved 97.6% relative content of ferrate(VI) (Fig. S2, Table S2).

### Probing the Reactivity Using C_60_ Molecules

We first probed the reactivity of K_2_FeO_4_ using C_60_. As a special kind of carbon material with defined molecular structure, C_60_ can provide directive and reliable assessment results. The spherical cage of C_60_ confers an excess of strain to C=C bonds, inducing a unique sp^2.28^ hybridization of the carbon atoms with a pyramidalization angle *θ*_P_ of 11.6° [44, 45]. This endows C_60_ with a moderate reactivity: inerter than that of the sp^3^-C atoms (*θ*_P_ = 19.5°) appearing as defects on carbon surface and more active than that of the sp^2^-C atoms with smaller *θ*_P_s in graphite (*θ*_P_ = 0°) and most other carbon materials (e.g., CNTs and CNFs) [46]. In addition, the small-molecule property of C_60_ allows its product structure to be easily determined by standard organic analytical methods such as mass and NMR spectroscopy.

**Fig. 2:**
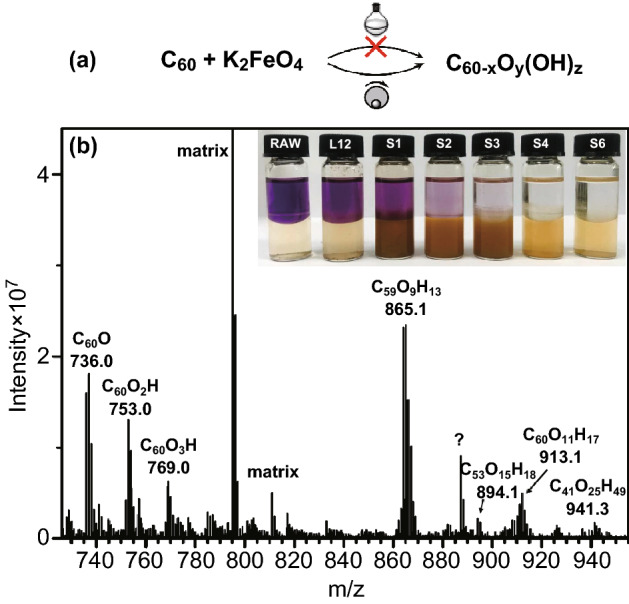
**a** Comparison of C_60_ oxidation results by K_2_FeO_4_ in liquid phase and solid state. **b** MS of oxidized C_60_ products by 6-h solid-state reaction (ball milling at 250 rpm). Inset shows photographs of C_60_ samples in toluene–water biphasic system. L12 refers to the product by 12-h liquid-phase treatment, and S1–6 refers to that by solid-state reaction for 1–6 h

After the liquid-phase oxidation, C_60_ products showed scarcely any changes with respect to the pristine sample, even with a large excess of K_2_FeO_4_ (ca. 300 mol equivalent) and long reaction time (up to 12 h). They displayed black color, poor water dispersibility and good solubility in toluene (Fig. 2b inset). The product structure was determined by MALDI-FTICR mass spectrometry (MS). Except from the prominent peak at m/z 720.000 (intact C_60_ ions), no newly produced ion peaks were observed (Fig. S3), which unambiguously showed K_2_FeO_4_ in liquid phase was not reactive enough to oxidize C_60_.

In contrast, K_2_FeO_4_ efficiently oxidized C_60_ in the “dry” way. By only 1 ~ 2-h treatment, the majority of products became hydrophilic, yielding a thick dark-brown aqueous layer after phase separation with toluene (Fig. 2b inset), which was ascribed to the generation of oxygen-containing groups. With prolonged reaction time, the purple toluene layer faded and the aqueous phase gradually became yellowish transparent, indicating continuous oxidative transformation and increasing oxidation degree [47]. The product MS showed many ion clusters above m/z 720.004, clearly separated by multiples of 16 (O) and 17 (OH) mass units. Various oxidized species were identified, including C_60_O (736.000), C_60_O_2_H (753.003), C_60_O_3_H (768.998) and C_60_O_11_H_17_ (913.078). The attachment of –OH groups onto C_60_ cage was also confirmed by ^1^H NMR (Fig. S4).

The oxidation treatment not only introduced oxygen and hydroxy entities to C_60_ cages, but also gave rise to cage-opened products such as C_59_O_9_H_13_ (865.057), C_53_O_15_H_18_ (894.063) and C_41_O_25_H_49_ (941.248). We suspected it was the mechanical force that broke the molecular cages, but the production of these broken cages was not remarkably depressed (62% vs. 50%, relative quantifications by MS) when the energy input was substantially decreased (from 250 rpm × 6 h to 180 rpm × 2 h). On the other hand, when K_2_FeO_4_ was replaced by a non-oxidizing isomorphous salt K_2_SO_4_, the amount of cage-opened products was dramatically reduced (20% by K_2_SO_4_ vs. 62% by K_2_FeO_4_, 250 rpm × 6 h). These results suggested that the intrinsic reactivity of K_2_FeO_4_ in solid state was strong enough to cleave the C=C bonds of C_60_ (presumably by the addition of Fe^VI^=O with C=C bonds).

Based on the results provided by C_60_ probe, it is clear that: (1) K_2_FeO_4_ in H_2_SO_4_ environment can hardly attack the *sp*^2.28^-C of strained C=C bonds. As a reasonable inference, it would not be able to oxidize the C=C bonds that are inerter in most carbon materials (such as the graphite in debate); (2) K_2_FeO_4_ in solid state can open the strained C=C bonds and even consume the C atoms in the skeleton. Therefore, the active defective sites on carbon surface could be readily oxidized by such dry chemistry, as has been observed in our previous work on DCNT functionalization [42].

### Further Testing the Reactivity Using Nanocarbons

It is still uncertain whether (1) the liquid-phase K_2_FeO_4_ takes effect in the oxidation of active defects and (2) solid-state K_2_FeO_4_ is oxidizing enough for less strained C=C bonds. To address these issues, the reactivity of K_2_FeO_4_ was further determined using nanocarbons including CNTs and CNFs, in both defective and graphitized types (Figs. 1c, S5 and S12). Specifically, DCNT contains rich numbers of surface defects (adatoms, vacancies, cracks, etc.) [48], exhibiting an *I*_D_/*I*_G_ ratio of up to 1.84, while GCNT has a well-graphitized, nearly defectless surface [49–51] with a very low *I*_D_/*I*_G_ of 0.028. DCNF is made of stacked graphene “cups” exposing large amounts of edge sites on the outer shells [52, 53] giving a high *I*_D_/*I*_G_ of 1.84, while GCNF, constructed by close packing of carbon rods, features edge-closed loops formed by high-temperature graphitization [54] with a much lower *I*_D_/*I*_G_ of 0.33.

#### K_2_FeO_4_ in Liquid Phase Could Only Oxidize Surface Defects

**Fig. 3:**
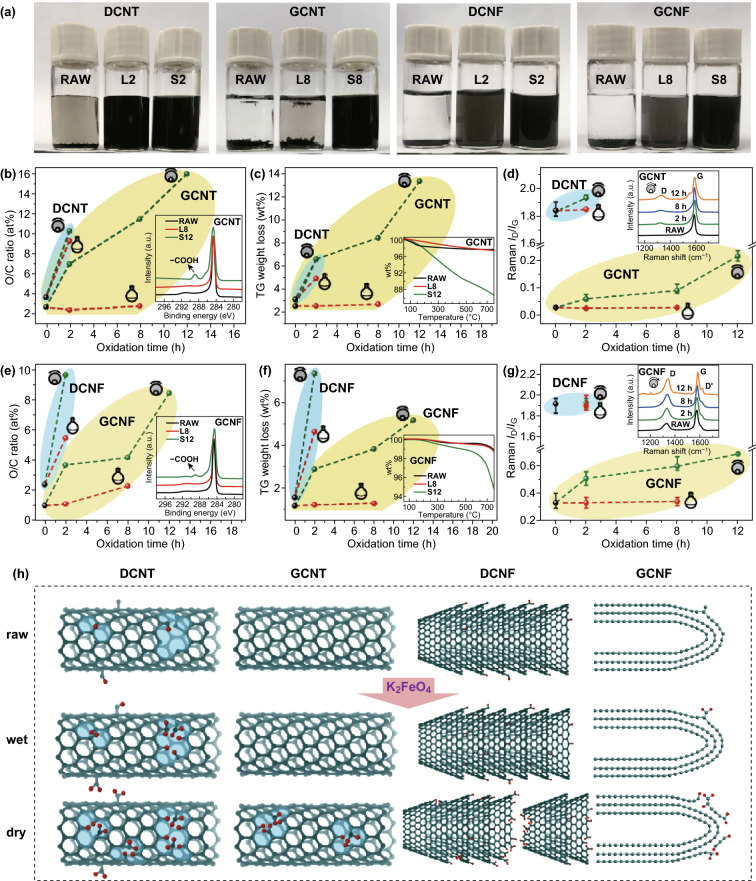
Oxidation results of various nanocarbons by K_2_FeO_4_ in liquid phase and solid state. If applicable, water-dispersible portions of the products were isolated for measurements to better show the oxidation effects. **a** Photographs of 0.1 mg mL^−1^ samples in water that had been sonicated for 5 min and settled for 3 days. **b**, **e** XPS O/C ratios (inset: representative C 1s spectra). **c**, **f** TG weight losses (inset: representative TG curves). **d**, **g** *I*_D_/*I*_G_ ratios (inset: representative Raman spectra). **h** A depiction of the effects of K_2_FeO_4_ oxidation on nanocarbons in liquid phase and solid state (oxygen atoms are shown in red). (Color figure online)

The oxidizing ability of K_2_FeO_4_ in liquid phase was found to be modest: the oxidation was efficient to the defective nanocarbons while inoperative to the graphitized ones. To be specific, DCNTs treated by K_2_FeO_4_/H_2_SO_4_ for 2 h showed good aqueous dispersibility, in contrast to the insoluble raw material (Fig. 3a). The surface O/C ratio (detected by XPS) showed an increase from 3.5% (raw) to 9.1% (2 h) (Fig. 3b). In addition, TG weight loss (Fig. 3c), originated from thermolysis of functional groups on surfaces, also supported the increased oxygen content on 2-h-treated DCNTs. However, GCNTs after treatment (up to 8 h) showed properties that were substantially unchanged compared to the raw material, including poor water dispersibility, few contents of surface oxygen and low levels of TG weight loss (Fig. 3a-c). The results of CNFs treated by K_2_FeO_4_ followed a similar trend with those of CNTs (Fig. 3a, e, f). Note that 8-h-treated GCNFs displayed distinguishable oxidation effects, since the so-called graphitized CNFs actually contained a certain number of defects in view of the *I*_D_/*I*_G_ of 0.33.

**Fig. 4:**
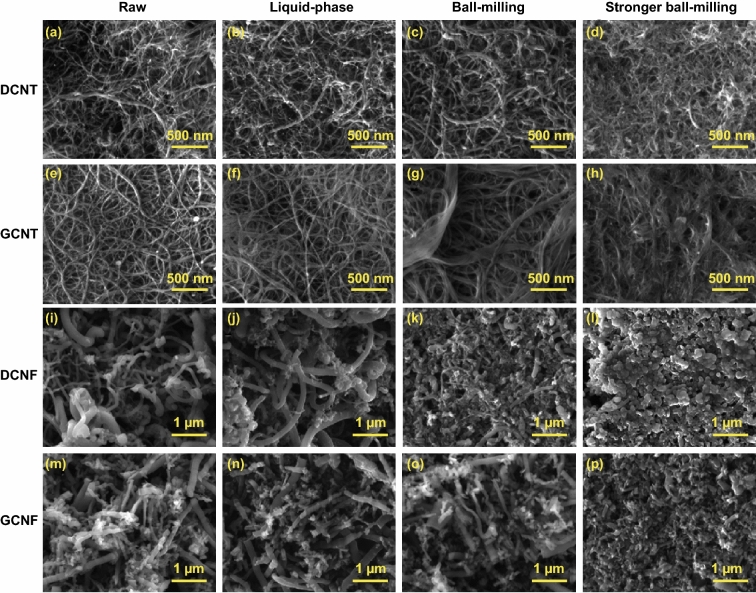
SEM images of carbon nanomaterials under different reaction conditions. **a–d** DCNTs: **a** raw, **b** L2, **c** S2 at 250 rpm, **d** S2 at 300 rpm; **e–h** GCNTs: **e** raw, **f** L8, **g** S8 at 250 rpm, **h** S8 at 300 rpm; **i-l** DCNFs: **i** raw, **j** L2, **k** S2 at 100 rpm, **l** S2 at 250 rpm; **m-p** GCNFs: **m** raw, **n** L8, **o** S2 at 100 rpm, **p** S12 at 100 rpm

The above results suggested that liquid-phase oxidation by K_2_FeO_4_ could only occur at the original defects on carbon materials. Raman spectra further confirmed that additional defects (which would arise from the reactions on C=C bonds) were not produced during the reaction process, as reflected by the almost unchanged *I*_D_/*I*_G_ values for both unoxidized nanocarbons and the oxidatively modified ones (Fig. 3d, g). As a result, carbon nanomaterials were protected from structural damage during the oxidation treatment, as shown by SEM and TEM images in Figs. 4 and S5. These results supported the nondestructive oxidation of CNTs reported by Zhang and Xu [39].

According to the reactivity clarified above, K_2_FeO_4_ in H_2_SO_4_ medium can only provide a slight oxidizing effect on graphite, which arises from reactions at the edge sites of graphene sheets, and the basal planes are unlikely to be affected. This explains why K_2_FeO_4_/H_2_SO_4_ method is unsuitable for preparing graphene oxide as pointed out by Sofer et al. [36]. On the other hand, when bulk graphite sample was replaced by nanographite platelets with high ratio of edge-to-plane sites (Fig. S6), distinct oxidizing effects were observed: the surface O/C ratio was increased to 6.8% with discernible -COOH peak in C 1s spectrum after 8-h treatment (Fig. S7), which manifested again the ability of K_2_FeO_4_ to oxidize the defective sites on carbon surface.

#### K_2_FeO_4_ in Solid State Could Open the Inert C=C Bonds

K_2_FeO_4_ in solid state was able to oxidize not only the defective nanocarbons but also the graphitized ones. For nanocarbons which got negligible oxidation in liquid phase, effective oxidative modifications were achieved in the solid state; and in cases when liquid-phase oxidation was successful, the solid-state reactions would provide higher levels of oxidation (Fig. 3, Table S4). Such strong oxidizing effect can be interpreted as the ability to open the inert C=C bonds in carbon lattice, as indicated by the evidently increased *I*_D_/*I*_G_ values after solid-state oxidations (Fig. 3d, g).

**Fig. 5:**
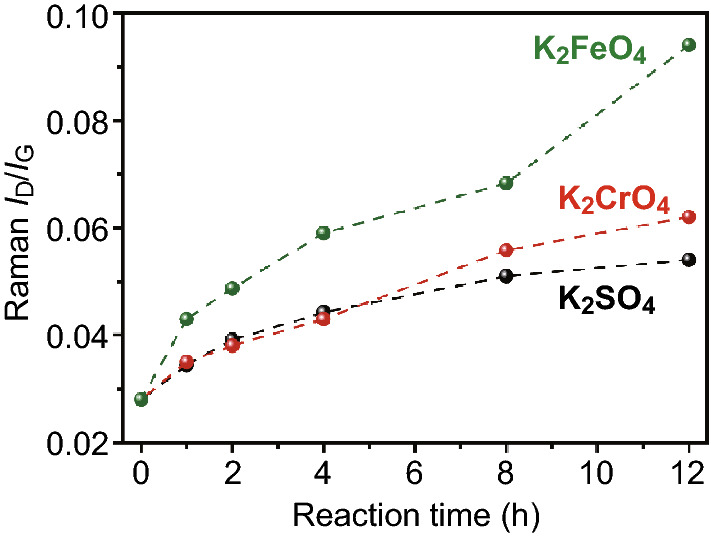
*I*_D_/*I*_*G*_ ratio results of GCNTs by solid-state milling (250 rpm) with three isomorphous salts. A comparison of Raman spectra is shown in Fig. S13

To figure out whether the bond breakage is merely a result of physical effect by ball milling or contributed from the chemical reactivity of K_2_FeO_4_ solids, two isomorphous salts, non-oxidizing K_2_SO_4_ and weakly oxidizing K_2_CrO_4_, were also used in the dry reaction system for comparison. We used a mild ball milling condition of 250 rpm as suggested by the C_60_ experiments, and the nearly defectless GCNTs were taken as substrate. As displayed in Fig. 5, GCNTs during ball milling with K_2_SO_4_ showed gentle increase in the defect content with time (*I*_D_/*I*_G_ only increased 0.025 after 12 h), which was reasonably induced by mechanical force. K_2_CrO_4_ treatment had a comparable effect with slightly more defects at long reaction times. In contrast, K_2_FeO_4_ led to efficient introduction of defects, producing much higher defect degrees than the other two salts at the same condition. Therefore, K_2_FeO_4_ solids possessed sufficient oxidizing ability to directly open the inert C=C bonds under the mechanochemical conditions. It was no surprise that C_60_ cages were easily damaged by such dry oxidation.

### Discussion

Our systematic studies using elaborately selected carbon materials clearly reveal two distinct oxidizing activities of ferrate(VI) depending on the reaction environment. Their different oxidation effects on four nanocarbons are depicted in Fig. 3h.

Liquid-phase oxidation only works for the defective sites, which means the reactivity of ferrate(VI) is largely depressed in H_2_SO_4_ medium and becomes much weaker than that of the commonly used oxidizers (e.g., KMnO_4_ and HNO_3_) for carbon materials. Such modest reactivity limits its scope of application, but may be desirable for functionalizing defective carbon materials, where effective surface oxidation can be achieved with no risk of disturbing the carbon structure or morphology.

In solid state, ferrate(VI) readily oxidizes the surface defects and its oxidizing power is strong enough to break the inert C=C bonds in carbon lattice. Solid-state ferrate(VI) oxidation is thus generally applicable for introducing oxygenated groups (e.g., −COOH) onto various carbon surface. In addition, the mechanical force involved in reaction can favor the oxidation performance by producing more defects. On the other hand, the mechanochemical conditions (e.g., rotation speed, ball type and time) must be optimized to avoid undesired structural damage, especially for fragile materials like CNFs (see Figs. 4 and S8–S11 for detailed studies on the effect of reaction condition on product structure).

## Conclusions

By using molecular and nanoforms of carbon as substrates, we unraveled the reactivity of ferrate(VI) in oxidizing carbon materials. The theoretically strong oxidizing power of ferrate(VI) is largely depressed in H_2_SO_4_ medium, yielding a modest reactivity that only oxidizes the active defects on carbon surface. This liquid-phase ferrate(VI) oxidation can be used as a gentle approach to functionalizing defect-rich carbon materials with the advantage of protecting structural integrity. Ferrate(VI) in solid state releases a high oxidizing power that is capable of opening the inert C=C bonds in carbon lattice, making it generally applicable to introduce oxygenated groups to various carbon materials. This intrinsic strong reactivity underlies the dry chemistry of ferrate(VI) and implies its wide scope of applications in green and powerful oxidative functionalization/transformation.

These two distinct oxidizing abilities could also apply to other kinds of materials. Considering the emerging role of high-valent iron compounds (represented by ferrate(VI)) in materials science, understanding their reactivity in different conditions is of fundamental importance for guiding their applications.

## Electronic supplementary material

Below is the link to the electronic supplementary material.

Supplementary material 1 (PDF 1944 kb)
