# Supplementary material for: Versatile Functionalization of Carbon Nanomaterials by Ferrate(VI)
Source: Nanomicro Lett. 2020 Jan 21;12:32. doi: 10.1007/s40820-019-0353-2 (PMC7770691; doi:10.1007/s40820-019-0353-2)
Supplement: Supplementary file 1 — Supplementary material 1 (PDF 1944 kb) [file 40820_2019_353_MOESM1_ESM.pdf]

Supporting Information for

Versatile Functionalization of Carbon Nanomaterials by Ferrate(VI)

Ying Zhou[1], Zhao-Yang Zhang[1,] *, Xianhui Huang[1], Jiantong Li[2], Tao Li[1,] *

[1]School of Chemistry and Chemical Engineering, Key Laboratory of Thin Film and Microfabrication (Ministry of Education), Shanghai Jiao Tong University, Shanghai 200240, People's Republic of China

[2]School of Electrical Engineering and Computer Science, KTH Royal Institute of Technology, Electrum 229, 16440 Kista, Sweden

*Corresponding authors. E-mail: zy_zhang@sjtu.edu.cn (Zhao-Yang Zhang); litao1983@sjtu.edu.cn (Tao Li)

S1 Supplementary Table

Table S1 Literature results on K_2FeO_4 oxidation of carbon materials in liquid phase

Refs.	carbon materials	oxidant	solvents	reaction condit	d_{002}[a] (nm)	O/C ratio[b] ('	I_D/I_G[c]
[S1]	1 g graphite (40 µm, ~0.6 µm)	6 g K_2FeO_4	40 mL cSA	1 h, RT	0.9 (0.34)	45.5	0.93
[S2]	10 g graphite (325 mesh)	9 g $KMnO_4$; 6 g K_2FeO_4	100 mL cSA	5 h, 35 °C	0.816 (0.34)	47.2	0.94
[S3]	10 g graphite (particle size <20µn	60 g K_2FeO_4	400 mL cSA	1 h, RT	0.35 (0.34)	18.3 (0)	0.23 (0.11)
[S4]	0.5 g graphite (120 mesh)	3 g K_2FeO_4	100 mL cSA + 6.6 mL cPA	36 h, 50 °C	lamellar layer gr	-	0.35 (0.156)
[S5]	100 mg CNTs	3 g K_2FeO_4	20 mL cSA	3 h, 60 °C	-	15.9 (6.5)	2.10 (2.06)
[S6]	1 g graphite (2-15 µm)	10 g K_2FeO_4	40 mL cSA	1 h, RT	0.34 (0.34)	2.0 (1.1)	Extremely low, almost the same as raw material

Note: cSA (concentrated sulfuric acid), cPA (concentrated phosphoric acid), RT (room temperature). Data of the raw materials are shown in parentheses.

[a] d_{002} represents the interlayer spacing of graphite, tested by XRD; [b] O/C ratio was tested by XPS; [c] I_D/I_G was tested by Raman spectra.

S2 Experimental Procedures

S2.1 Preparation of K_2FeO_4

S2.1.1 Chemicals

The main chemicals for synthesis and purification of K_2FeO_4 are hydrochloric acid (AR, 37%, Sinopharm), potassium permanganate (AR, Sinopharm), potassium hydroxide (GR, 95%, Macklin), n-pentane (AR, 99%, Aladdin), tetrahydrofuran (AR, 99%, Lingfeng), isopropanol (ACS, 99.5%, Aladdin), absolute ethanol (pharmaceutical grade, 99.5%, Dr. Mao), anhydrous methanol (AR, 99.5%, Sinopharm), anhydrous diethyl ether (AR, 99.5%, Lingfeng). Ultrapure water (18.2 MΩ.cm) was used throughout the experiments.

S2.1.2 Synthesis and Purification Procedure

Preparation procedure of K_2FeO_4 was adapted from literature [7]. 205 mL of 37% HCl was slowly added dropwise to 33.4 g of $KMnO_4$ to produce Cl_2. Cl_2 was bubbled through the pre-chilled KOH solution (60 g of KOH in 100 mL of water) for about 2 h to synthesize KClO. Then, 90 g of KOH was added to the solution and the resulted suspension was cooled to 0 °C with stirring for 1 h. KCl formed as a byproduct and was removed by filtration. The leaving yellow solution of KClO was cooled to 0 °C and then 37.5 g of $Fe(NO_3)_3 \cdot 9H_2O$ was slowly added to the solution with rapid stirring for 2 h. Then, 50 mL of 3 M KOH was poured into the solution with standing for 15 min. The obtained dark purple suspension was washed with 3 M KOH and filtered to get the solution of the K_2FeO_4. Later, solid KOH was slowly added to the solution to form a saturated solution under cooling conditions (<5 °C) to separate out K_2FeO_4. The resulting solution was allowed to stand for 40 min and then filtered. The filtrate was discarded and the precipitate was washed successively with n-pentane, tetrahydrofuran, isopropanol, absolute ethanol, anhydrous methanol and anhydrous diethyl ether. Then it was dried at 45 °C in a vacuum oven.

The synthesized K_2FeO_4 was further purified by recrystallization as described in our previous work [S8].

S2.1.3 Characterization

The purity of K_2FeO_4 was determined by **UV-Vis spectrophotometry** [9]. using molar absorptivity $\varepsilon_{510\ nm}$ = 1150 M^{-1} cm^{-1} of FeO_4^{2-} in 5 mM Na_2HPO_4/1 mM $Na_2B_4O_7$ buffer (pH 9.20) [8]. **XRD** patterns were obtained on a Bruker D8 Advance diffractometer (Cu Kα radiation, λ=1.54184 nm) and data were recorded in the 2θ range of 10-80° at 6 °/min; The **transmission ^{57}Fe Mössbauer spectroscopy** was performed by using a Mössbauer spectrometer in a constant acceleration mode with a ^{57}Co(Pd) source (Wissel, Germany).

S2.2 Oxidation Treatment

S2.2.1 Materials and Chemicals

Fullerene C_{60} (purity > 99.5%) was purchased from TCI (Shanghai) Development Co., Ltd. DCNT is muti-walled tube between 8 and 15 nm in outer diameter with lengths of 50 μm (Chengdu Organic Co. Ltd., Chinese Academy of Sciences, China). GCNT is few-walled tube (1-3 layers) with outer

diameter less than 2 nm and lengths of 5-30 μm (Chengdu Organic Co. Ltd., Chinese Academy of Sciences, China). DCNF is herringbone type fiber between 50 and 200 nm in diameter with lengths of 10-30 μm (product code: GNF-100; Carbon Nano-material Technology Co., LDT, Korea). GCNF is formed by heat treatment of platelet graphite nanofibers with average diameter of 80 nm (product name: Carbon Nanochips; Catalytic Materials LLC, USA). Nanographite (D50 < 400 nm, Macklin). Sulfuric acid (AR, Sinopharm), potassium sulfate (AR, Sinopharm), potassium chromate (AR, Sinopharm).

S2.2.2 Liquid-phase K_2FeO_4 Oxidation

Oxidation of DCNT

100 mg of DCNTs was slowly added to 20.0 mL of sulfuric acid (95%-98%) in a 50 mL two-necked flask under argon atmosphere, and the dispersion was sonicated for 30 min. Then 2.5 g of K_2FeO_4 was slowly added to the flask under argon flow at 0°C, and the reaction mixture was stirred at 60 °C for 2 h. The resulting dispersion was diluted in 500 mL of cold water and settled for 30 min. The solid was obtained by centrifugation (5000 rpm, 10 min), followed by washing with 2 M HCl (several times to remove Fe^{3+}). The obtained solid was then dispersed in water under brief sonication, followed by filtration (0.22 μm PTFE membrane) and extensive washing with water to become neutral. Water-dispersible portions of the DCNTs were isolated as follows. The products were redispersed in water under sonication for 10 min, followed by centrifugation at 5000 rpm for 10 min to get the supernatant. Then the sediment was collected together to repeat the process of redispersion/centrifugation till the supernatant became clear. After that, the collected black supernatant was precipitated by 1 M HCl, followed by filtration and successive washing with water, alcohol and diethyl ether. The obtained product was finally dried at 60 °C in a vacuum oven.

S2.2.3 Oxidation of GCNT

100 mg of GCNTs was slowly added to 20.0 mL of sulfuric acid (95%-98%) in a 50 mL two-necked flask under argon atmosphere, and the dispersion was sonicated for 30 min. Then, 2.5 g of K_2FeO_4 was slowly added to the flask under argon flow at 0 °C, and the reaction mixture was stirred at 60 °C for 2 or 8 h. The resulting dispersion was diluted in 500 mL of cold water and settled for 30 min. The solid was obtained by centrifugation (5000 rpm, 10 min), followed by washing with 2 M HCl (several times to remove Fe^{3+}). The obtained solid was then dispersed in water under brief sonication, followed by filtration (0.22 μm PTFE membrane) and extensive washing with water, alcohol and diethyl ether. The obtained product was finally dried at 60 °C in a vacuum oven.

S2.2.4 Oxidation of DCNF

100 mg of DCNFs was slowly added to 20.0 mL of sulfuric acid (95%-98%) in a 50 mL two-necked flask under argon atmosphere. After that, 2.5 g of K_2FeO_4 was slowly added to the flask under argon flow at 0 °C, and the reaction mixture was stirred at 60 °C for 2 h. The resulting dispersion was diluted in 500 mL of cold water and settled for 30 min. The solid was obtained by centrifugation (5000 rpm, 10 min), followed by washing with 2 M HCl (several times to remove Fe^{3+}). The obtained solid was then dispersed in water under brief sonication, followed by filtration (0.22 μm PTFE membrane) and extensive washing with water to become neutral. Water-dispersible portions of the

DCNFs were isolated as follows. The products were redispersed in water under sonication for 10 min, followed by centrifugation at 3000 rpm for 10 min to get the supernatant. Then the sediment was collected together to repeat the process of redispersion/centrifugation till the supernatant became clear. After that, the collected black supernatant was precipitated by 1 M HCl, followed by filtration and successive washing with water, alcohol and diethyl ether. The obtained product was finally dried at 60 °C in a vacuum oven.

S2.2.5 Oxidation of GCNF

100 mg of GCNFs was slowly added to 20.0 mL of sulfuric acid (95%-98%) in a 50 mL two-necked flask under argon atmosphere. After that, 2.5 g of K_2FeO_4 was slowly added to the flask under argon flow at 0 °C, and the reaction mixture was stirred at 60 °C for 2 or 8 h. The resulting dispersion was diluted in 500 mL of cold water and settled for 30 min. The solid was obtained by centrifugation (5000 rpm, 10 min), followed by washing with 2 M HCl (several times to remove Fe^{3+}). The obtained solid was then dispersed in water under brief sonication, followed by filtration (0.22 μm PTFE membrane) and extensive washing with water, alcohol and diethyl ether. The obtained product was finally dried at 60 °C in a vacuum oven.

S2.2.6 Oxidation of Nanographite

The nanographite used in this experiment was purified from the commercial nanographite. The commercial nanographite was dispersed in DMSO and sonicated for 30 min to form a dispersion of 1 mg mL^{-1}. Then, the black supernatant was collected by centrifuging at 4000 rpm for 15 min, while the bulk precipitates were discarded. The dispersed nanographite in the supernatant was collected by centrifuging at 12,000 rpm for 5 min. The obtained sample was finally dried at 60 °C in a vacuum oven.

100 mg of purified nanographite was slowly added to 20.0 mL of sulfuric acid (95%-98%) in a 50 mL two-necked flask under argon atmosphere. After that, 2.5 g of K_2FeO_4 was slowly added to the flask under argon flow at 0°C. Next, the dispersion was stirred at 60 °C for 2 h or 8 h or 12 h. The resulting dispersion was diluted in 500 mL of cold water and settled for 30 min. The solid was obtained by centrifugation (5000 rpm, 10 min), followed by washing with 2 M HCl (several times to remove Fe^{3+}). The obtained solid was then dispersed in water under brief sonication, followed by filtration (0.22 μm PTFE membrane) and extensive washing with water to become neutral. Water-dispersible portions of the nanographite were isolated as follows. The products were redispersed in water under sonication for 10 min, followed by centrifugation at 5000 rpm for 10 min to get the supernatant. Then the sediment was collected together to repeat the process of redispersion/centrifugation till the supernatant became clear. After that, the collected black supernatant was precipitated by 1 M HCl, followed by filtration and successive washing with water, alcohol and diethyl ether. The obtained product was finally dried at 60 °C in a vacuum oven.

S2. 3 Solid-state K_2FeO_4 Oxidation

S2.3.1 Oxidation of DCNT

100 mg of DCNTs and 2.5 g of K_2FeO_4 were mixed together by brief grinding in an agate mortar. The

mixture was then introduced into a 50 mL stainless milling jar together with 26 g of 5 mm-diameter stainless steel balls (ball-to-powder weight ratio 10:1). Ball milling was performed at a rotational speed of 250 or 300 rpm for 2 h in a horizontal planetary ball milling (WXQM-2L, Tecan Powder). The jar was opened every 30 min to break up the mixture materials if they were agglomerated or adhered to the sidewall during milling process. The resulting mixture was slowly added to 50 mL of 2 M HCl and settled for 2 h, followed by centrifugation at 5000 rpm for 10 min to get the solid. The acid washing process was repeated for 3 times to completely remove the ferric irons. The obtained solid was filtrated and washed by water to become neutral. Water-dispersible portions of the DCNTs were isolated as follows. The products were redispersed in water under sonication for 10 min, followed by centrifugation at 5000 rpm for 10 min to get the supernatant. Then the sediment was collected together to repeat the process of redispersion/centrifugation till the supernatant became clear. After that, the collected black supernatant was precipitated by 1 M HCl, followed by filtration and successive washing with water, alcohol and diethyl ether. The obtained product was finally dried at 60°C in a vacuum oven.

S2.3.2 Oxidation of GCNT

100 mg of GCNTs and 2.5 g of K_2FeO_4 were mixed together by brief grinding in an agate mortar. The mixture was then introduced into a 50 mL stainless milling jar together with 26 g of 5 mm-diameter balls (stainless steel balls). Ball milling was performed at a rotation speed of 250 or 300 rpm for different reaction time (2, 8 or 12 h). The jar was opened every 1 h to break up the mixture materials if they were agglomerated or adhered to the sidewall during milling process. The resulting mixture was slowly added to 50 mL of 2 M HCl and settled for 2 h, followed by centrifugation at 5000 rpm for 10 min to get the solid. The acid washing process was repeated for 3 times to completely remove the ferric irons. The obtained solid was filtrated and washed by water to become neutral. If applicable, water-dispersible portions of the GCNTs were isolated as follows. The products were redispersed in water under sonication for 10 min, followed by centrifugation at 5000 rpm for 10 min to get the supernatant. Then the sediment was collected together to repeat the process of redispersion/centrifugation till the supernatant became clear. After that, the collected black supernatant was precipitated by 1 M HCl, followed by filtration and successive washing with water, alcohol and diethyl ether. The obtained product was finally dried at 60 °C in a vacuum oven.

S2.3.3 Oxidation of DCNF

100 mg of DCNFs and 2.5 g of K_2FeO_4 were mixed together by brief grinding in an agate. The mixture was then introduced into a 50 mL stainless milling jar together with 26 g of 5 mm-diameter balls (stainless steel balls or agate balls). Ball milling was performed at different rotational speeds including 100, 150, and 250 rpm for 2 h. The jar was opened every 30 min to break up the mixture materials if they were agglomerated or adhered to the sidewall during milling process. The resulting mixture was slowly added to 50 mL of 2 M HCl and settled for 2 h, followed by centrifugation at 5000 rpm for 10 min to get the solid. The acid washing process was repeated for 3 times to completely remove the ferric irons. The obtained solid was filtrated and washed by water to become neutral. Water-dispersible portions of the DCNFs were isolated as follows. The products were redispersed in water under sonication for 10 min, followed by centrifugation at 3000 rpm for 10 min

to get the supernatant. Then the sediment was collected together to repeat the process of redispersion/centrifugation till the supernatant became clear. After that, the collected black supernatant was precipitated by 1 M HCl, followed by filtration and successive washing with water, alcohol and diethyl ether. The obtained product was finally dried at 60 °C in a vacuum oven.

S2.3.4 Oxidation of GCNF

100 mg of GCNF and 2.5 g of K_2FeO_4 were mixed together by brief grinding in an agate mortar. The mixture was then introduced into a 50 mL stainless milling jar together with 26 g of 5 mm-diameter balls (stainless steel balls or agate balls). Ball milling was performed at a rotational speed of 100 or 250 rpm for different reaction time including 2, 8, and 12 h. The jar was opened every 1 h to break up the mixture materials if they were agglomerated or adhered to the sidewall during milling process. The resulting mixture was slowly added to 50 mL of 2 M HCl and settled for 2 h, followed by centrifugation at 5000 rpm for 10 min to get the solid. The acid washing process was repeated for 3 times to completely remove the ferric irons. The obtained solid was filtrated and washed by water to become neutral. If applicable, water-dispersible portions of the GCNFs were isolated as follows. The products were redispersed in water under sonication for 10 min, followed by centrifugation at 3000 rpm for 10 min to get the supernatant. Then the sediment was collected together to repeat the process of redispersion/centrifugation till the supernatant became clear. After that, the collected black supernatant was precipitated by 1 M HCl, followed by filtration and successive washing with water, alcohol and diethyl ether. The obtained product was finally dried at 60°C in a vacuum oven.

S2.4 Characterization

For **MALDI-FT ICR MS** analysis, a Bruker 7.0 T SolariX FTICR hybrid quadrupole-FT ICR mass spectrometer (Bruker Daltonics, Bremen, Germany), equipped with an ESI/APCI/MALDI ion source, and external ion accumulation was used in positive ion mode over a mass range of m/z 150.48-3000 in broadband detection mode. This MALDI source has a pulsed smart beam-II UV laser with an attenuator that allows fine adjustment of laser fluence (Azura Laser AG, Berlin, Germany). The MS instrument was tuned and calibrated with sodium trifluoroacetate in ESI source. The laser beam focus was set at the small value. The detector plate was 210 V, plate offset was 80 V, laser power was 25%-35%, frequency was 500 Hz; laser shots were 250, time of light was 0.9 ms. **^{1}H NMR** (400 MHz) spectroscopic data were recorded on an AVANCE III HD 400 spectrometer with chemical shift of the residual solvent as an internal reference. **Raman spectra** were recorded with a 633 nm excitation laser by using a DXR Raman microscope (Thermo Fisher Scientific). **XPS** measurements were performed by ESCALAB 250Xi X-ray photoelectron spectroscopy (Thermal Scientific) using Al Kα X-rays and the binding energies were calibrated with respect to C 1s peak at 284.6 eV. **TG** was measured on a Pyris 1 thermogravimetric analyzer (TA), in which the temperature was increased to 700 °C at a heating rate of 10 °C /min under nitrogen. **SEM** images were acquired using a MIRA3 instrument (TESCAN). **TEM** was conducted on Tecnai G2 spirit Biotwin (FEI) and TALOS F200X (FEI) transmission electron microscopes.

S2.5 Quantification of Cage-opened C_{60}

The product of C_{60} was dissolved in toluene under sonication for 5 min, followed by centrifugation at

12000 rpm for 3 min to remove the unreacted C_{60} in the supernatant. Then the obtained sediment was subjected to repeated process of dissolution/centrifugation till the supernatant became colorless. The sample was then dried at 40 °C in a vacuum oven and The sample were tested by MALDI-FT ICR MS and relative content of the cage-opened products was quantified by Eq. S1:

$$\mu = (m_1 - m_2 + m_3 \cdot \alpha)/m_1 \qquad \text{(S1)}$$

where μ is the relative content of cage-opened products, m_1 is the mass of raw C_{60}, m_2 is the mass of C_{60} product after reaction, m_3 is the mass of sediment after wash by toluene, α represents the relative percentage of cage-opened products in MALDI-FT ICR MS spectrum.

S3 Results and Discussion

S3.1 Purity Analysis of K_2FeO_4

Our K_2FeO_4 has a purity of 95%, as determined by spectrophotometry. The XRD pattern showed diffraction peaks that matches well with the standard PDF card of K_2FeO_4 crystals (Fig. S1), confirming the single-phase character of the sample without any crystalline impurities (e.g., KCl, KNO_3, and $K_2CO_3 \cdot 1.5H_2O$). The purity of ferrate(VI) was also determined by the ^{57}Fe Mössbauer spectrum. As shown in Fig. S2, the value of the isomer shift parameter at room temperature (-0.89 mm s^{-1}) corresponds well with the previously reported data for K_2FeO_4 [S10, S11] and the relative content of Fe(VI) was up to 97.6%, proving the high purity of our potassium ferrate(VI) again.

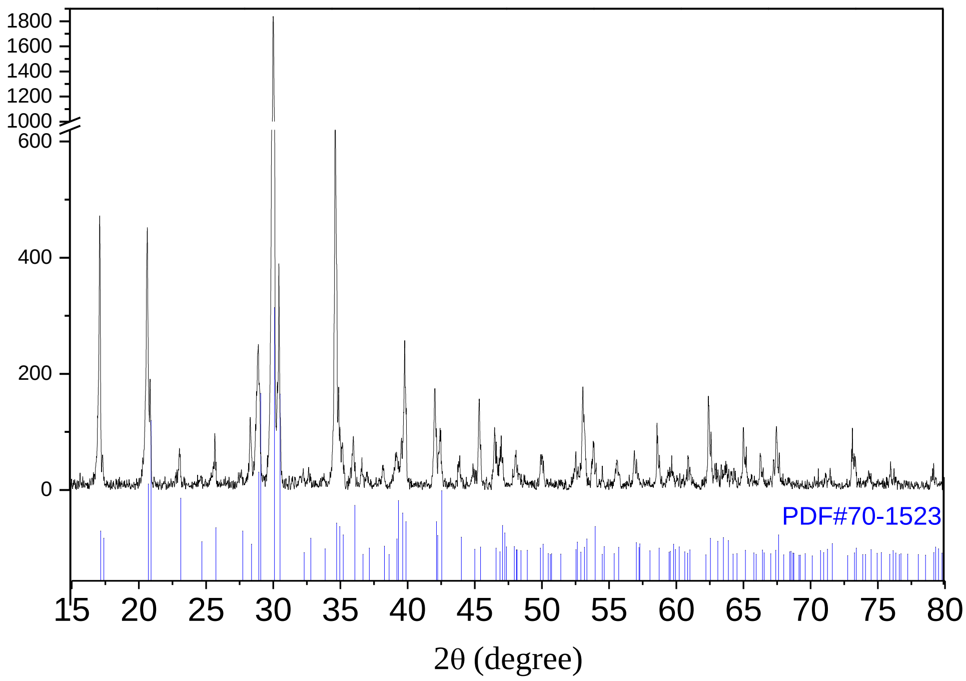

Fig. S1 A comparison of XRD patterns of K_2FeO_4 sample and the standard PDF card

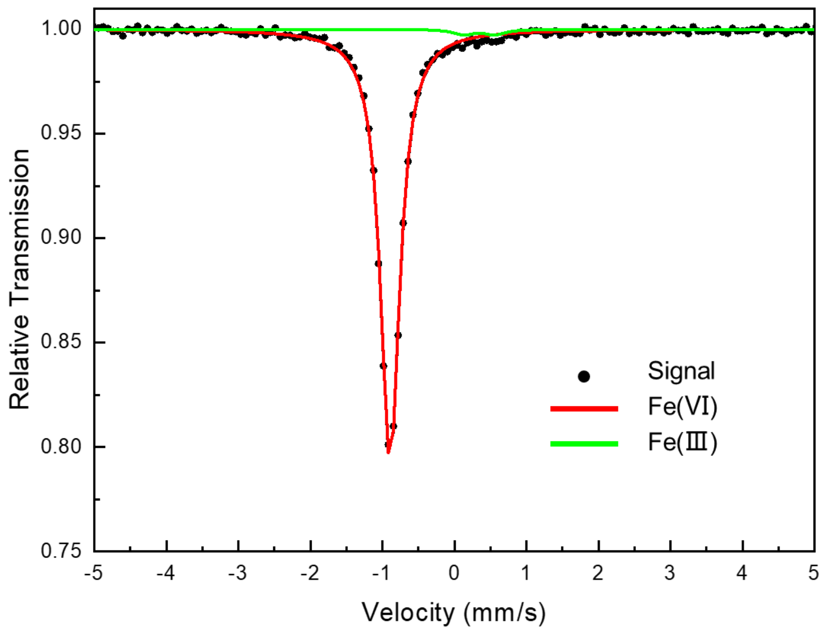

Fig. S2 [57]Fe Mössbauer spectrum of K_2FeO_4 sample

Table S2 [57]Fe Mössbauer spectroscopy results of K_2FeO_4 sample

materials	isomer shift (mm/s)	quadrupole splitting (mm/s)	FWHM (mm/s)	relative area (%)
Fe(VI)	-0.89	0	0.16	97.6
Fe(III)	0.34	0.43	0.17	2.4

S3.2 Oxidation of C_{60}

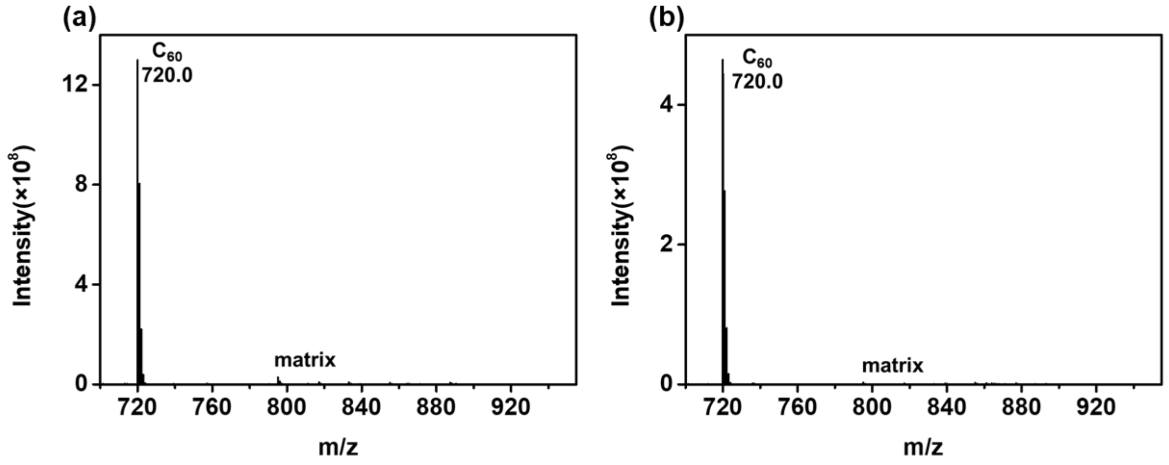

Fig. S3 MALDI-FT ICR MS of (a) pristine C_{60} and (b) C_{60} after treated by K_2FeO_4/H_2SO_4 for 12 h

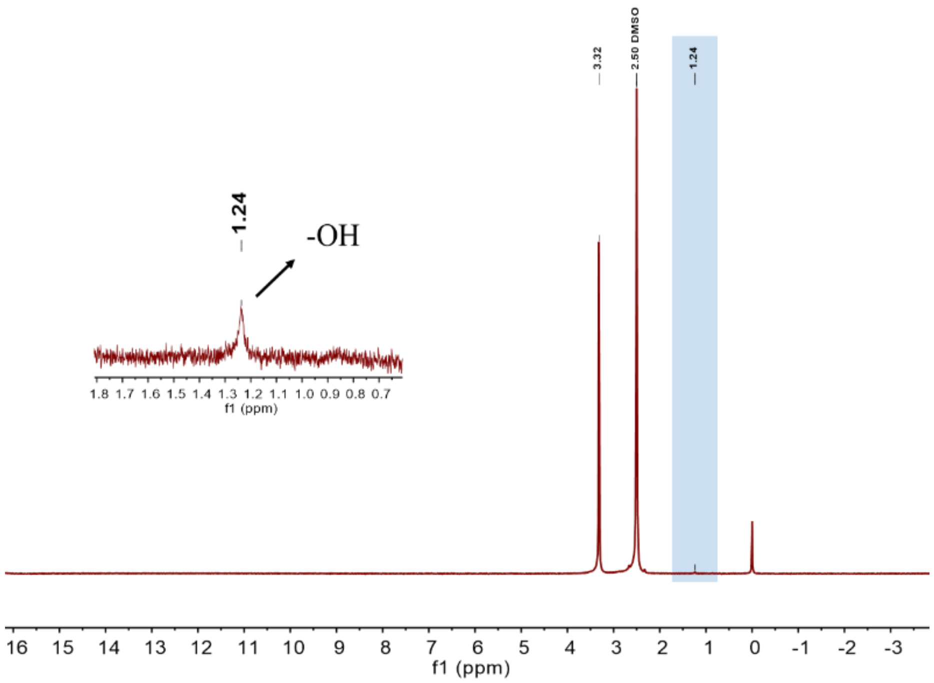

Fig. S4 ^{1}H NMR spectrum of oxidized C_{60} (DMSO-d_6, 400 MHz)

S3.3 Oxidation of Carbon Materials

S3.3.1 Liquid-phase Oxidation

We note that the amount of K_2FeO_4 have an influence on the oxidation performance. In liquid phase oxidation, the dosage of 1.5 g and 2.5 g respectively led to 28.6% and 37.4% yield of the water-soluble DCNT product.

2.5 g dosage per 100 mg of carbon materials were used throughout our experiments.

As shown in Fig. S5, the morphology of the nanocarbons was not affected by the liquid-phase oxidation. Large amounts of long individual tubes or fibers were clearly observed in the products (even with a long reaction time), which was consistent with the pristine materials. Therefore, K_2FeO_4 in H_2SO_4 environment is mild enough for protection of the carbon structures.

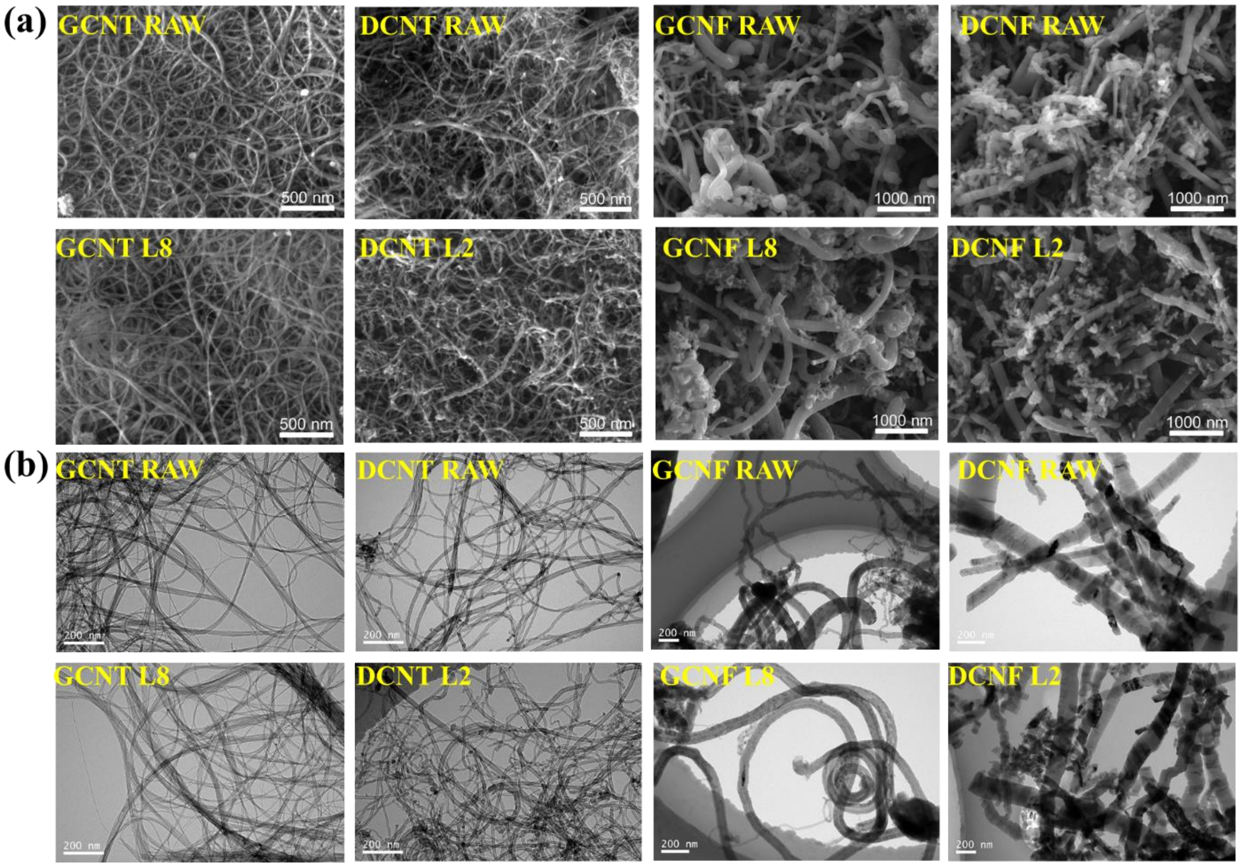

Fig. S5 (**a**) SEM and (**b**) TEM images of four typical nanocarbons before and after liquid-phase reaction

The commercial nanographite consisted of a number of different-sized platelets and many of them were in several microns, as shown in Figure S6a. We have extracted the smaller platelets with lateral sizes well below 1 μm (Fig. S6b), which provide much higher ratio of edge-to-plane sites for our experiment.

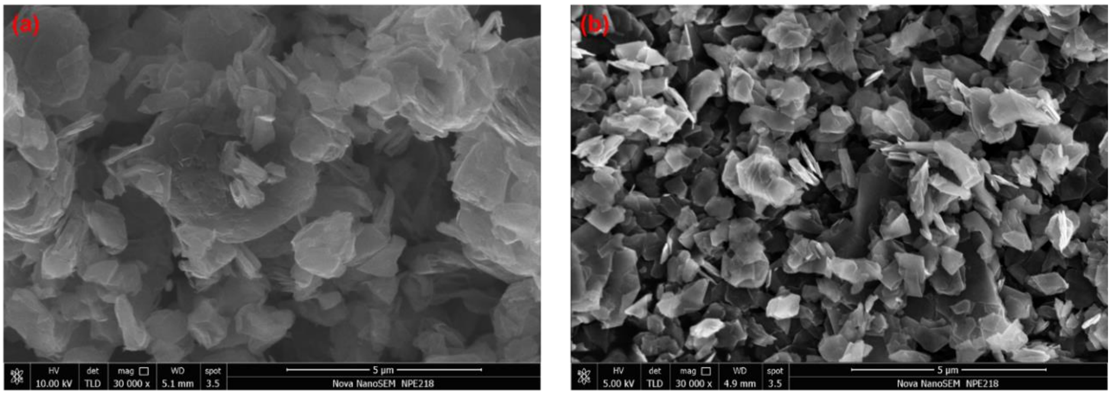

Fig. S6 SEM images of (**a**) commercial and (**b**) separated nanographite

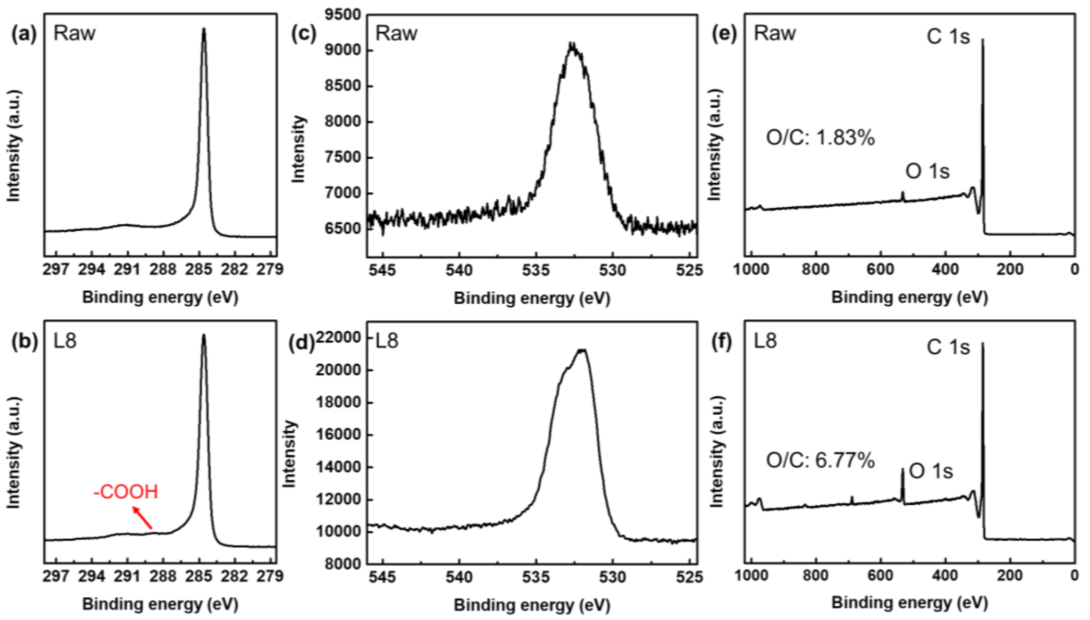

Fig. S7 XPS results of nanographite before and after K_2FeO_4/H_2SO_4 treatment for 8 h (**a, b**) C 1s spectra, (**c, d**) O 1s spectra, (**e**, **f**) XPS survey spectra

S3.3.2 Solid-state Oxidation

The strength of milling force can be evaluated by stress energy SE, given by Eq. S2

$$SE = \rho \cdot v_t^2 \cdot d^3 \qquad (S2)$$

where v_t is the circumferential velocity, ρ and d is the density and diameter of the milling ball, respectively [S12]. In our experiments, the diameter of the milling ball was fixed at 5 mm, and the influence factors were rotation 1speed and the density/type of the milling ball.

As shown in Fig. S8, raw DCNTs exhibited tangled network consisted of long tubes. After 2 h-ball-milling treatment, the morphology of DCNTs remained similar features at the speed of 250 rpm. However, when the SE was increased by 1.44-fold (300 rpm), the structural integrity of DCNTs were destroyed, resulting in shortened and curled tubes.

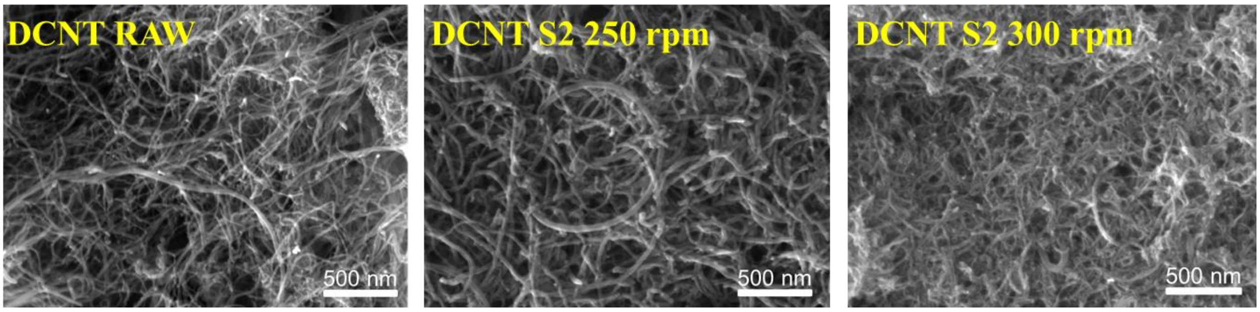

Figure S8 SEM images of DCNTs before and after milling under different conditions (stainless steel balls were used)

Similar phenomena occurred for GCNTs (Fig. S9). No obvious damage was observed at the speed of

250 rpm, indicating that 250 rpm provide mild milling strength for GCNTs. When the input energy was stronger at 300 rpm, severe structural damage was observed.

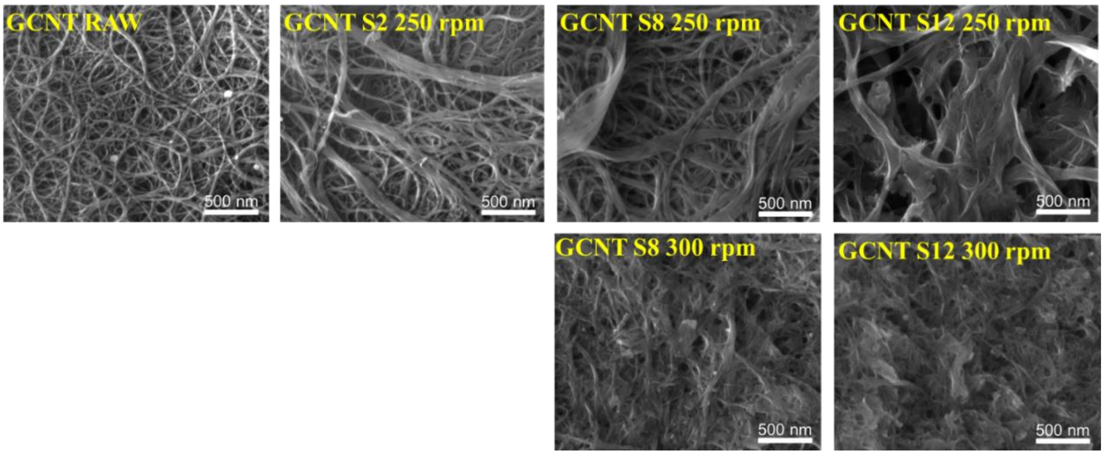

Fig. S9 SEM images of GCNTs before and after milling under different conditions (stainless steel balls were used)

For fragile GCNFs (Fig. S10), we suggested the use of low-density agate balls, mild milling speed (100 rpm) and short reaction time (2 h) to protect the integrity of the fibers. When the milling time was increased, GCNFs were damaged gradually under these mild conditions. On the other hand, the fibers were quickly destroyed when high-density stainless steel balls were used with a higher milling speed (250 rpm).

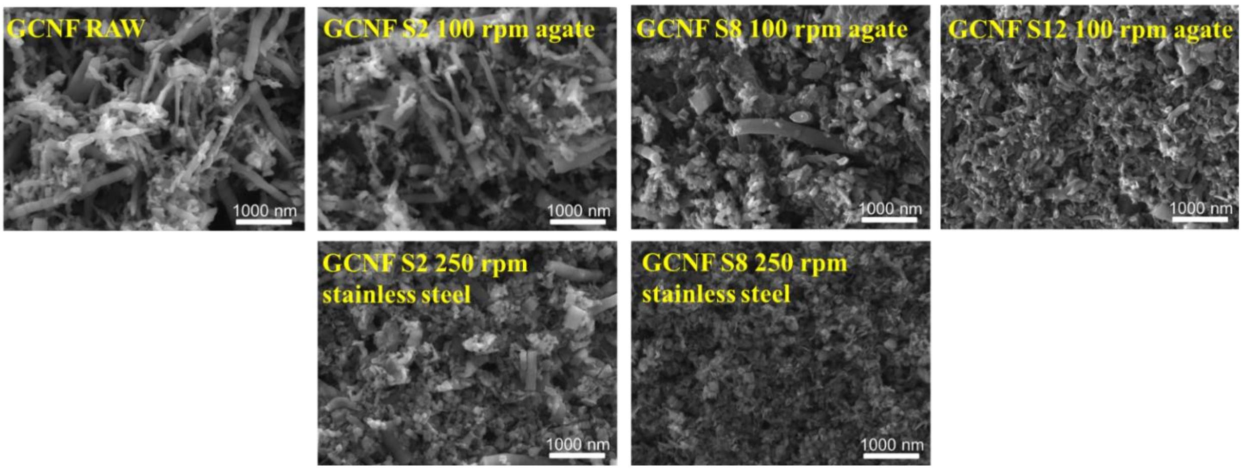

Fig. S10 SEM images of GCNFs before and after milling under different conditions

For DCNFs that were even more fragile (Fig. S11), mild energy input (100 rpm× 2 h, agate balls) could also cause structural damage. Any further increase of the milling speed or the density of milling balls could make the situations worse: most of the fibers were cut short and even became fragments.

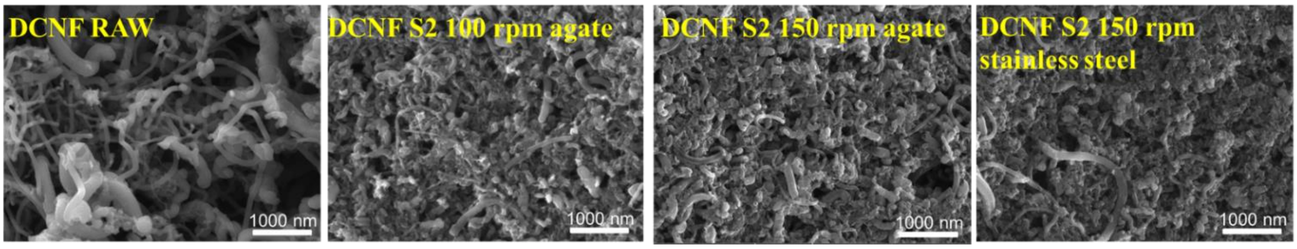

Fig. S11 SEM images of DCNFs before and after milling under different conditions

The HRTEM images of nanocarbons in Fig. S12 further confirmed that the structures of these carbon materials remained intact by liquid-phase reaction and the mild mechanochemical conditions.

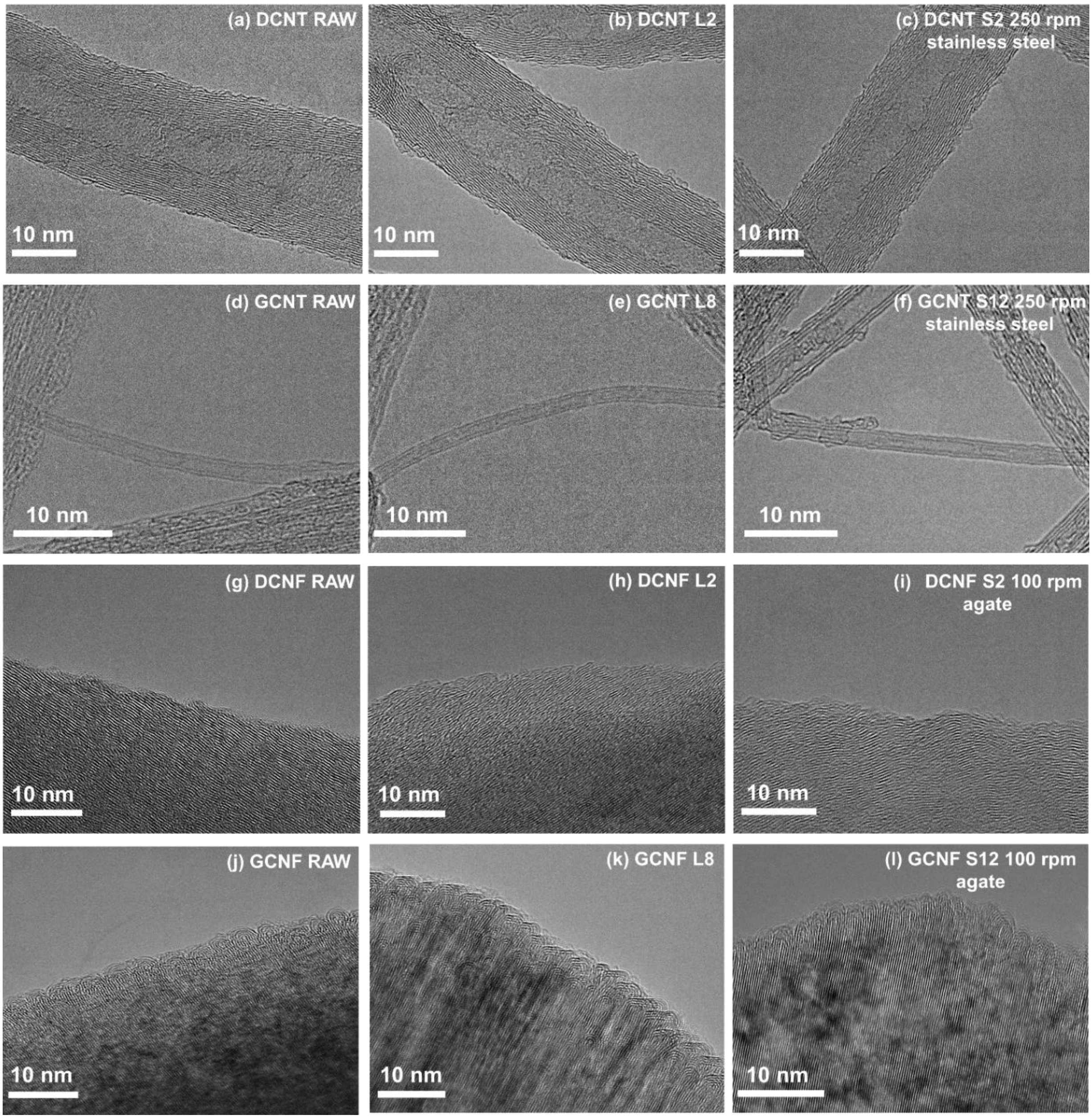

Fig. S12 HRTEM images of four typical nanocarbons before and after oxidations

Table S3 The structural information of the three isomorphous salts

Chemical Formula	PDF Card	Space Group	ɑ	b	c
K2SO4	05-0613	Pmcn (62)	5.772	10.072	7.483
K2FeO4	70-1523	Pnma (62)	7.694	5.858	10.360
K2CrO4	15-0365	Pnam (62)	7.663	10.391	5.919

After K_2FeO_4 oxidation, obvious increase in I_D/I_G was observed due to the opening of C=C bonds. It also led to large upshifts in D and G bands, arising from the "doping" effect by oxygenated groups [S13, S14]. Compared to K_2SO_4, K_2CrO_4 only led to slight changes in the Raman spectra, because its ability to open C=C bonds is much weaker than that of K_2FeO_4.

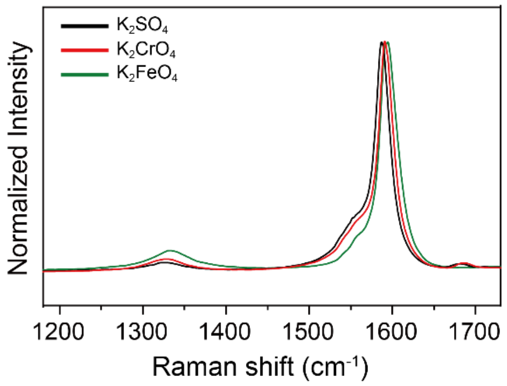

Fig. S13 Comparison of the Raman spectra of 12 h products. To compare the overall defect degree, water-dispersible portions of the products were not isolated, thus lower I_D/I_G ratios were recorded than those in Figure 3d for the K_2FeO_4 samples.

S3.3.3 Comparison of Liquid-phase and Solid-state Oxidation

Table S4 Comparison of the oxidation degree under different reaction conditions

Materials	Properties	Raw	Liquid phase		Solid state [a]		
			2 h	8 h	2 h	8 h	12 h
DCNT	O/C ratio (%)	3.5	9.1	--	10.1	--	--
	TG weight loss (%)	3.0	4.8	--	6.6	--	--
GCNT	O/C ratio (%)	2.5	2.2	2.6	6.8	10.3	15.8
	TG weight loss (%)	2.6	2.8	2.7	6.5	8.3	13.3
DCNF	O/C ratio (%)	2.4	5.5	--	8.9	--	--
	TG weight loss (%)	1.6	4.6	--	7.4	--	--
GCNF	O/C ratio (%)	1.0	1.1	2.3	3.7	4.4	8.5
	TG weight loss (%)	1.1	1.2	1.3	2.9	3.8	5.2

[a] 250 rpm using steel balls for DCNT and GCNT; 100 rpm using agate balls for DCNF and GCNF

Supplementary References

[1] L. Peng, Z. Xu, Z. Liu, Y. Wei, H. Sun, Z. Li, X. Zhao, C. Gao, An iron-based green approach to 1-h production of single-layer graphene oxide. Nat. Commun. **6**, 5716 (2015). http://dx.doi.org/10.1038/ncomms6716

[2] H. Yu, B. Zhang, C. Bulin, R. Li, R. Xing, High-efficient synthesis of graphene oxide based on improved hummers method. Sci. Rep. **6**, 36143 (2016). http://dx.doi.org/10.1038/srep36143

[3] A. Romero, M.P. Lavin-Lopez, L. Sanchez-Silva, J.L. Valverde, A. Paton-Carrero, Comparative study of different scalable routes to synthesize graphene oxide and reduced graphene oxide. Mater. Chem. Phys. **203**, 284-292 (2018). http://dx.doi.org/10.1016/j.matchemphys.2017.10.013

[4] S. Mura, Y. Jiang, I. Vassalini, A. Gianoncelli, I. Alessandri et al., Graphene oxide/iron oxide nanocomposites for water remediation. ACS Appl. Nano Mater. **1**(12), 6724-6732 (2018). http://dx.doi.org/10.1021/acsanm.8b01540

[5] Z. Y. Zhang and X. C. Xu, Nondestructive covalent functionalization of carbon nanotubes by selective oxidation of the original defects with K_2FeO_4. Appl. Surf. Sci. **346**, 520-527 (2015). http://dx.doi.org/10.1016/j.apsusc.2015.04.026

[6] Z. Sofer, J. Luxa, O. Jankovsky, D. Sedmidubsky, T. Bystron, M. Pumera, Synthesis of graphene oxide by oxidation of graphite with Ferrate(VI) compounds: myth or reality? Angew. Chem. Int. Ed. **55**(39), 11965-9 (2016). http://dx.doi.org/10.1002/anie.201603496

[7] C. Li, X. Z. Li, N. Graham, A study of the preparation and reactivity of potassium ferrate. Chemosphere. **61**(4), 537-43 (2005). http://dx.doi.org/10.1016/j.chemosphere.2005.02.027

[8] Z.Y. Zhang, D. Ji, W. Mao, Y. Cui, Q. Wang et al., Dry chemistry of Ferrate(VI): A solvent-free mechanochemical way for versatile green oxidation. Angew. Chem. Int. Ed. **57**, 10949 (2018). http://dx.doi.org/10.1002/anie.201805998

[9] Z. Luo, M. Strouse, J.Q. Jiang, V.K. Sharma, Methodologies for the analytical determination of ferrate(VI): a review, J. Environ. Sci. Heal. A. **46**(5), 453-60 (2011). http://dx.doi.org/10.1080/10934529.2011.551723

[10] G.K. Wertheim, R.H. Herber, Resonant gamma - ray absorption in potassium ferrate. J. Chem. Phys. **36**(9), 2497-2499 (1962). http://dx.doi.org/10.1063/1.1732914

[11] L. Machala, R. Zboril, V.K. Sharma, J. Filip, D. Jancik, Z. Homonnay, Transformation of solid potassium ferrate(VI) (K_2FeO_4): mechanism and kinetic effect of air humidity. Eur. J. Inorg. Chem. **2009**(8), 1060-1067 (2009). http://dx.doi.org/10.1002/ejic.200801068

[12] C. Damm, T.J. Nacken, W. Peukert, Quantitative evaluation of delamination of graphite by wet media milling. Carbon. **81**, 284-294 (2015). http://dx.doi.org/10.1016/j.carbon.2014.09.059

[13] K.Y. Chun, I.K. Moon, J.H. Han, S.H. Do, J.S. Lee, S.Y. Jeon, Highly water-soluble multi-walled carbon nanotubes amine-functionalized by supercritical water oxidation. Nanoscale **5**(21), 10171-4 (2013). http://dx.doi.org/10.1039/c3nr03784c

[14] G.U. Sumanasekera, J.L. Allen, S.L. Fang, A.L. Loper, A.M. Rao, P.C. Eklund, Electrochemical oxidation of single wall carbon nanotube bundles in sulfuric acid. J. Phys. Chem. B. **103**(21), 4292-4297 (1999). http://dx.doi.org/10.1021/jp984362t
